# Extremely Premature Infant and Digestive Malformations: Case Report of Atypical Postoperative Journeys

**DOI:** 10.3390/pediatric17050101

**Published:** 2025-10-01

**Authors:** Elena Roxana Matran, Alexandru Dinulescu, Ana Prejmereanu, Oana-Alexandra Peta, Radu-Ioan Tiron, Mirela Luminița Pavelescu

**Affiliations:** 1 Department of Pediatrics, Faculty of Medicine, “Carol Davila” University of Medicine and Pharmacy, 050474 Bucharest, Romania; elena.smadeanu@umfcd.ro (E.R.M.); ana.prejmereanu@rez.umfcd.ro (A.P.); oana-alexandra.bogdan@rez.umfcd.ro (O.-A.P.); radu-ioan.tiron@rez.umfcd.ro (R.-I.T.); mirela.pavelescu@umfcd.ro (M.L.P.); 2 Department of Pediatrics, Emergency Hospital for Children “Grigore Alexandrescu”, 011743 Bucharest, Romania; 3 Department of Pediatrics, Children’s Clinical Hospital Dr. Victor Gomoiu, 022102 Bucharest, Romania; 4 Department of Pediatrics, National Institute for Mother and Child Health “Alessandrescu-Rusescu”, 010024 Bucharest, Romania

**Keywords:** extremely preterm infant, intestinal obstruction, colostomy, case report, microcolon

## Abstract

Background and Clinical Significance: Extremely premature infants face complex medical challenges requiring comprehensive multidisciplinary care. Gastrointestinal malformations, while rare, pose significant diagnostic and therapeutic challenges in this vulnerable population. Case Presentation: We report a case of an extremely premature infant born at 26 weeks gestation with very low birth weight (950 g) who developed a digestive pathology rarely encountered in neonatal intensive care: microcolon, which required surgical consultation and intervention, followed by an atypical postoperative course. Conclusions: The early recognition of gastrointestinal malformations in extremely premature infants requires high clinical suspicion and prompt multidisciplinary intervention. Despite complex postoperative course, favorable outcomes are achievable with coordinated care.

## 1. Introduction and Clinical Significance

Extreme prematurity is associated with numerous risks and complications. Due to the immaturity of all organs and systems, extremely low-birth-weight (ELBW) infants are a unique group of patients in the neonatal intensive care unit (NICU) [1,2,3]. They are extremely sensitive to small changes in respiratory management, blood pressure, fluid administration, nutrition, and virtually all other aspects of care [1]. Research continues to determine the optimal way to care for these infants [1].

Preterm birth is a heterogeneous complex syndrome with different etiologies and outcomes, requiring classification according to the underlying pathophysiology [4]. Understanding the specific etiology of preterm birth is crucial for predicting complications and planning appropriate interventions. In our country, there is a high mortality rate due to delivery before 28 weeks of gestation [2]. Preterm infants represent a challenge not only for neonatologists but also for the whole health system [5,6]. The most important aspects of protocols may be the uniformity of approach within an institution and a commitment to provide and evaluate care in a collaborative manner across professional disciplines.

Gastrointestinal complications in extremely premature infants range from feeding intolerance to rare congenital malformations such as microcolon. The early recognition and appropriate management of these conditions are critical for favorable outcomes [7,8].

This case report presents the successful surgical management of microcolon in an extremely premature infant weighing < 1000 g, demonstrating the importance of multidisciplinary care and early intervention.

## 2. Case Presentation

### 2.1. Prenatal History and Delivery

We report a case of an extremely preterm male newborn born at 26 weeks gestational age, with very low birth weight approximately 950 g, delivered by cesarean section, from cephalic presentation at a tertiary care maternity hospital from Bucharest, Romania. The newborn came from a high-risk pregnancy, obtained naturally.

The pregnancy was high-risk with multiple complications:

- Oligohydramnios (likely secondary to prolonged rupture of membranes).
- Maternal SARS-CoV-2 infection.
- Maternal hypothyroidism.
- Positive Escherichia coli culture.
- Membranes ruptured > 48 h before delivery.

The etiology of preterm birth in this case was likely infectious/inflammatory due to the prolonged rupture of membranes and maternal infection.

### 2.2. Initial Assessment and Resuscitation

Physical examination revealed poor general condition with generalized cyanosis and shallow breaths for which alveolar recruitment was performed using a Neopuff system. The infant was intubated in the delivery room, and the skin became pink with a subsequent improvement in color; he had rhythmic heart sounds, no murmurs, diminished tone and reactivity, absent archaic reflexes, no obvious congenital malformations detected clinically, and normotensive anterior fontanelle. The infant obtained an Apgar score of 5 at 1 min, 6 at 5 min, and 8 at 10 min, having reserved prognosis. During resuscitation in the delivery room, the newborn presented oxygen saturations according to a Flow Diagram for the first 10 min of life, being resuscitated (alveolar recruitment) with FiO2 30%. After 10 min of life, the SpO2 level was 96–98% at fraction of inspired oxygen (FiO2) 30%.

### 2.3. NICU Course—First Two Weeks

The newborn was admitted to the neonatal intensive care unit where he was placed at the point of thermal neutrality (closed incubator with servo control, T = 37 °C, humidity 80%), and mechanical ventilation was initiated in a high-frequency oscillation (HFO) system, with hydroelectrolytic and acid–base balancing infusion, Caffeine loading dose, and antibiotic therapy: Meropenem, Vancomycin, and Dopamine in the mesenteric/renal dose. Table 1 summarizes the key clinical milestones during the initial hospitalization.

### 2.4. Gastrointestinal Complications and Diagnosis

From a digestive point of view, the newborn presented a supple, depressible abdomen and eliminated meconium, and trophic feeding was initiated after 48 h of life (0.5 mL amino acid-based hypoallergenic formula every 6 h, then 3 h later, 1 mL). The abdomen remained supple, depressible, without vomiting, without gastric residue, and without stool in the last 48 h before the surgical consultation.

**Figure 1:**
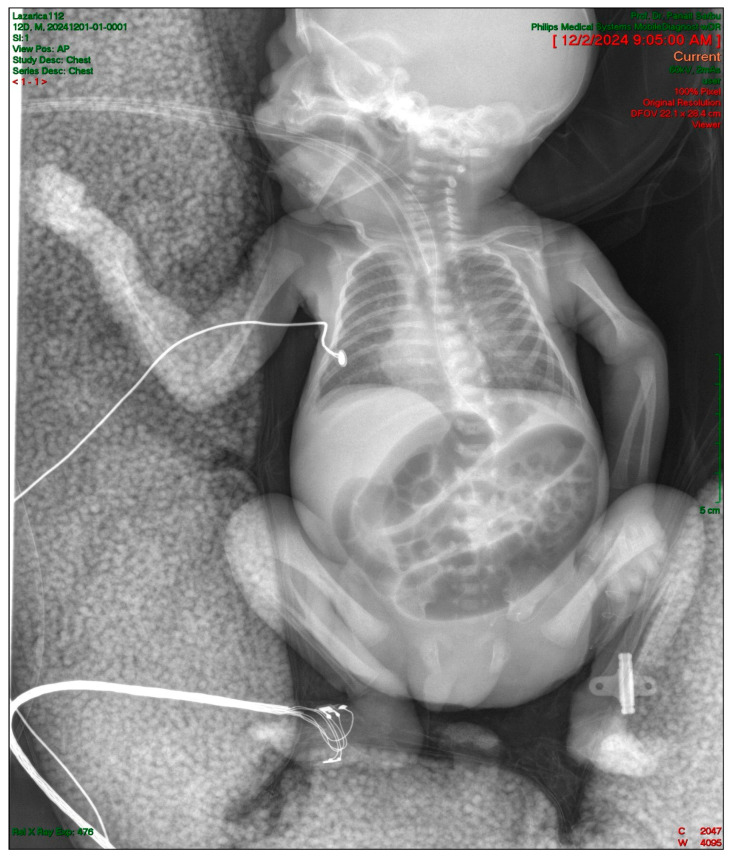
Abdominal X-ray demonstrating significantly dilated intestinal loops, with absence of gas in pelvic region, suggesting distal intestinal obstruction. This radiographic finding, combined with clinical symptoms of feeding intolerance and absent stool passage, prompted urgent surgical consultation for suspected intestinal obstruction.

An abdominal ultrasound was performed which detected a round, inhomogeneous mass with a diameter of 1.7 cm at the level of the ileocecal junction, with intestinal peristalsis present. Abdominal X-ray showed very dilated loops, without aeration in the pelvic region (Figure 1).

**Figure 2:**
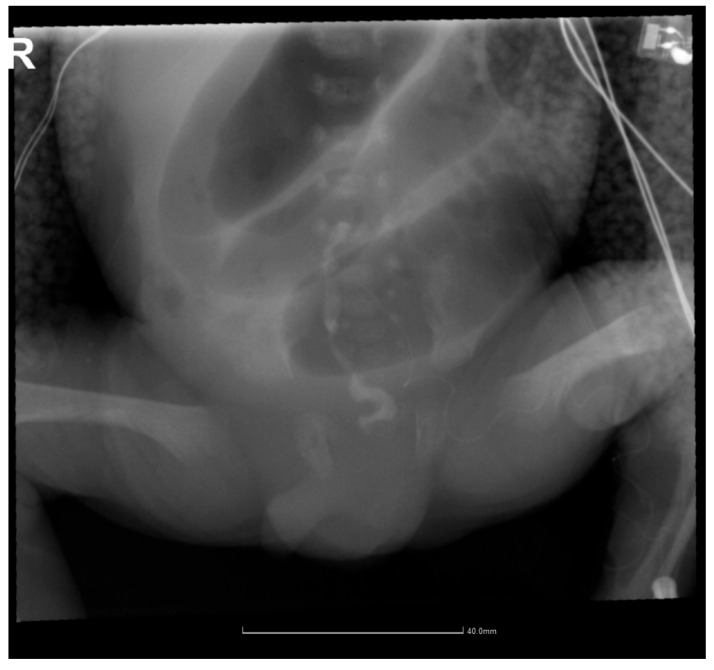
A contrast enema demonstrating microcolon. The distal unused colon appears significantly smaller in caliber compared to the markedly dilated proximal bowel, confirming the diagnosis of functional intestinal obstruction. This imaging finding led to the surgical decision for colostomy creation (R = right).

An enema with warm physiological serum was attempted, but the probe penetrated the anus 0.5–1 cm, using a gentle clockwise massage, glycerin suppository, and digestive rest. On the 12th day of life, intestinal obstruction was suspected, and he was sent for a surgical consultation. Contrast enema with Iomeron revealed microcolon (Figure 2). Surgical intervention was decided, and colostomy (sigmoidostomy) was performed using a “double-barrel” technique, with the probe maintained between the external distal end and the rectum. The operative findings revealed normal small bowel from the Treitz angle to the ileocecal valve, with the colon at the sigmoid level in a “finger-like” dilatation. The distal sigmoid appeared hypoplastic (approximately 0.4 cm diameter) but with anal patency confirmed.

### 2.5. Postoperative Course and Complications

Postoperative evolution: extubated on postoperative day 5, ventilated on nasal SIMV, Non-invasive ventilation continued for 48 h, favorable respiratory evolution, cardiorespiratory and hemodynamic stability maintained. Enteral feeding was reinitiated via gastroclysis (postoperative day 8), with intestinal transit through colostomy observed.

Genetic and pathological consultations ruled out megacystis-microcolon syndrome. Complete urological evaluation was performed given the association between microcolon and genitourinary abnormalities, revealing normal kidney function and bladder anatomy.

### 2.6. Long-Term Follow-Up and Complications

The infant developed bronchopulmonary dysplasia and retinopathy of prematurity requiring laser therapy. He was discharged at 40 weeks corrected gestational age, weighing 2340 g. In evolution, after discharge, the premature infant was clinically evaluated by the neonatologist and surgeon, the evolution being favorable; the weight curve was ascending, he received bottle feeding well, intestinal transit was present on the colostomy, and he was well cared for at home.

At the age of 4 chronological months (corrected age—1 month), the child was admitted to the pediatric department for evaluation, because he presented psychomotor agitation during defecation, and a thoraco-abdominal radiography indicated respiratory complications. This episode required hospitalization for bacterial infection of digestive origin and protein–calorie malnutrition, treated successfully with antibiotics and nutritional support.

**Figure 3:**
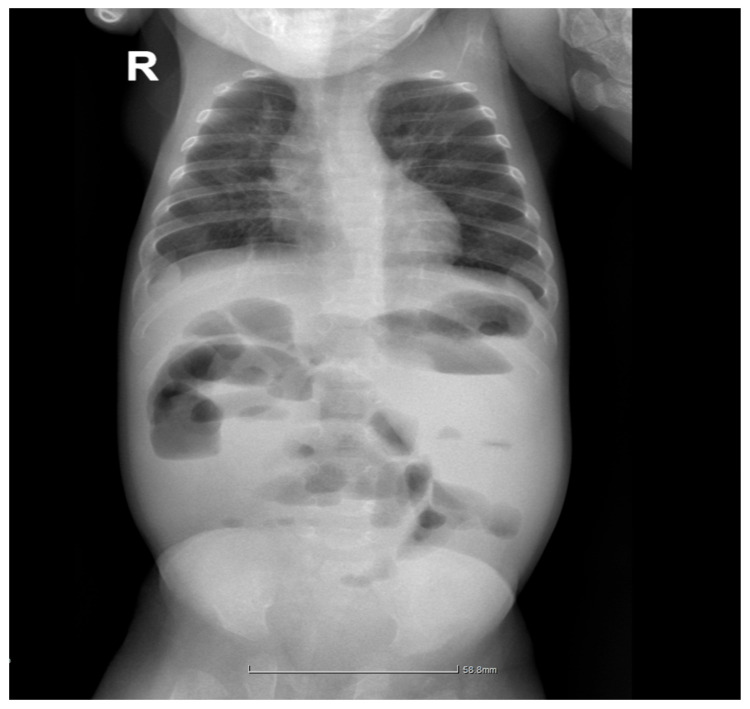
Preoperative abdominal X-ray showing dilated bowel loops and air–fluid levels in the ascending and transverse colon, indicating colostomy dysfunction. These findings necessitated surgical intervention with colostomy closure and end-to-end colo-colic anastomosis (R = right).

At approximately 4 months and 3 weeks of chronological age, the infant was admitted for colostomy dysfunction requiring surgical intervention. The preoperative abdominal X-ray showed several hydroaerial images projected in the upper and central half of the abdominal area corresponding to the ascending and transverse colon (Figure 3). Colostomy closure was decided upon and performed with end-to-end colo-colic anastomosis, viscerolysis, and perianastomotic peritoneal drainage. The postoperative evolution was favorable, with the infant being discharged in good general condition.

Subsequently, the infant underwent a follow-up program in the maternity ward but also with the surgeon and pediatrician, monitoring the weight curve, diet, nutritional status, and prevention of respiratory complications. Thus, Synagis was administered monthly to prevent respiratory syncytial virus infection; additional iron and vitamin D3 were administered; antireflux milk formula was administered.

### 2.7. Current Status and Long-Term Outcomes

**Figure 4:**
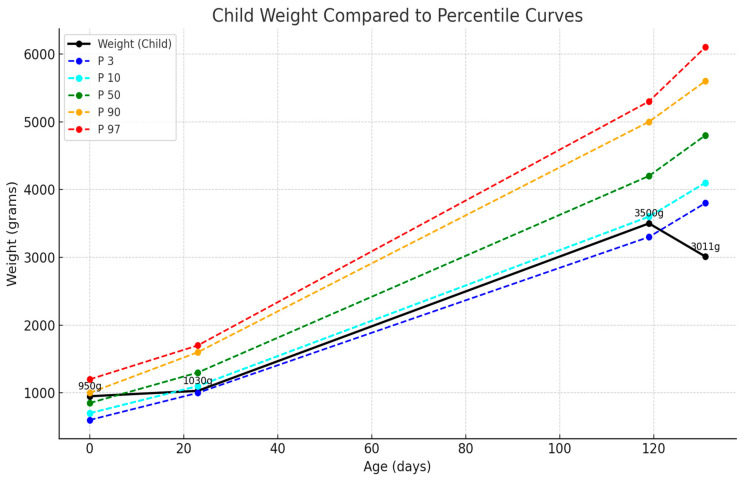
Growth trajectory showing patient weight compared to Fenton preterm growth chart percentiles from birth to 6 months chronological age. Despite initial complications and surgical interventions, the infant demonstrates appropriate catch-up growth with weight progression following expected curves for extremely premature infants.

This case of extreme prematurity had a favorable evolution despite the comorbidities, and the infant underwent successful surgical intervention at <1000 g without neurological sequelae. At 6 months chronological age (approximately 2 months corrected age), the infant demonstrated appropriate growth and development. Figure 4 shows weight progression compared to preterm growth chart percentiles, indicating catch-up growth despite initial complications [9]. At the time of this article’s conception, the infant made significant medical progress and is now under the care of a neonatologist for follow-up in the maternity ward. He was 6 months old medically and approximately 2 months old corrected.

## 3. Discussion

### 3.1. Unique Aspects of This Case

This case presents several atypical features that distinguish it from typical microcolon presentations: (1) extremely low birth weight at time of surgery (<1000 g), (2) early colostomy closure requirement due to dysfunction, and (3) successful outcome despite multiple complications.

### 3.2. Literature Review

Digestive pathology in extremely premature infants represents a challenge for the entire medical team. Microcolon is rarely encountered in neonatal intensive care, particularly requiring surgical intervention at birth weights below 1000 g. The evolution from the digestive perspective has revealed unique and challenging aspects for neonatologists.

The literature specifically addressing microcolon in very-low-birth-weight infants remains limited, reflecting the extreme rarity of this condition in this population. Early recognition came from a 1987 case series published by Amodio, Berdon, and Stolar that described six premature infants with birth weights ranging from 920 to 1320 g who developed marked abdominal distension with contrast enema examination revealing microcolon. Four of these infants were born to mothers with toxemia who received magnesium sulfate, and notably, bilious emesis was absent despite marked distension. This study established microcolon of prematurity as a distinct form of functional obstruction [10].

Emil et al. (2004) subsequently provided more structured guidelines, reporting meconium obstruction in ELBW neonates weighing less than 1500 g, with an incidence of 1.5% in this population [11]. This foundational work demonstrated the feasibility of surgical intervention in extremely premature infants and established early management protocols. More recently, Solaz-García et al. (2019) characterized prevention strategies for meconium obstruction in very-low-birth-weight preterm infants, representing one of the few contemporary studies addressing this specific population [12]. Most published cases involve term or near-term infants with isolated microcolon or as part of megacystis-microcolon syndrome [13,14,15,16].

While specific microcolon literature is limited, the broader understanding of gastrointestinal complications in ELBW infants has expanded significantly. The colon is a relatively uncommon site for intestinal atresia, with an estimated incidence of 5–15% of all intestinal atresia in neonates [17]. Bowel obstruction represents one of the most common surgical emergencies in newborns, with an estimated incidence of 1 in 2000 live births [18].

Contemporary studies have focused primarily on more common gastrointestinal pathologies in ELBW infants, particularly necrotizing enterocolitis and spontaneous intestinal perforation, which have become increasingly recognized as the predominant surgical bowel diseases in this population [19,20,21]. These studies provide valuable context for understanding surgical outcomes and decision-making in ELBW infants, even though they do not specifically address microcolon.

The scarcity of the literature specific to microcolon in ELBW infants underscores the exceptional nature of our case. Recent advances in neonatal intensive care have improved outcomes for ELBW infants with gastrointestinal complications generally, but the specific management of microcolon at birth weights below 1000 g remains largely guided by individual case experience and extrapolation from related conditions [22,23].

The differential diagnosis of microcolon includes functional immaturity versus structural abnormalities such as megacystis-microcolon syndrome, which was appropriately ruled out through genetic consultation in our case [24,25]. The management approach requires individualized decision-making based on the infant’s overall clinical condition, gestational age, and institutional expertise.

The limited literature available highlights the need for multicenter case series and registries to better characterize the presentation, management, and outcomes of microcolon in extremely premature infants. Our case contributes to this sparse but important body of knowledge.

During hospitalization in the neonatal intensive care unit, the infant presented with PDA. This pathology has been observed in premature infants, especially those from mothers with hypothyroidism, the thyroid hormone being essential for the normal closure of the ductus arteriosus after birth [26,27,28].

The role of maternal COVID-19 infection in preterm birth has been increasingly recognized. In our case, maternal infection combined with prolonged membrane rupture likely contributed to extremely preterm delivery. Recent systematic reviews demonstrate increased preterm birth risk with maternal COVID-19: Allotey et al. (2020), OR 1.57 (95% CI 1.36–1.81) for preterm birth; SET-NET surveillance data, 12.9% preterm birth rate in infected mothers; English cohort data: higher very preterm birth rates (<32 weeks) [29,30,31]. Pathophysiological mechanisms involve direct viral effects and maternal inflammatory responses, causing placental changes that may trigger preterm labor [32,33,34].

Another challenge in this case was the prevention of fungal infections; the premature neonate received antibiotic treatment and was hospitalized for extended periods in intensive care. Sepsis remains difficult to diagnose owing to its non-specific clinical manifestations, underscoring the necessity for prompt detection. Additionally, preventing fungal infections presents another challenge; premature neonates are often administered antibiotics and require extended hospitalization in the intensive care unit [35,36].

### 3.3. Nutritional Considerations

Ensuring adequate nutrition in this case was complicated by the following: (1) initial feeding intolerance due to intestinal obstruction, (2) postoperative feeding restrictions, (3) recurrent infections affecting nutritional status, and (4) the need for specialized formulas post-colostomy. The development of protein–calorie malnutrition required targeted nutritional intervention and monitoring. Ensuring sufficient and optimal nutrition for preterm infants in the neonatal intensive care unit presents a significant challenge, both during hospitalization and after discharge. Experts in the field have to consider both digestive tolerance and potential digestive malformations or uncommon complications. The main health problems of premature babies after discharge from the maternity hospital are as follows: chronic lung disease, growth retardation/protein–calorie malnutrition, and infections [37]. Children born preterm are more likely to have growth delays compared with those born at term [38]. In a follow-up study of 950 children born with low birth weight (<1500 g), infants with persistent poor growth at 9 and 24 months (length for age Z-scores < −2) had lower motor and cognitive scores on the Bayley Scales of Infant Development than those with better growth measures (length for age Z-scores > −2) [38,39]. From a neurological point of view, the evolution of our case in the first 6 months of life was favorable but requires further evaluation. Our patient presented, in the context of the digestive pathology—colostomy—both infectious risks and extrauterine growth restriction and gastroesophageal reflux/regurgitation, specific to extreme prematurity and accentuated by the atypical complication, requiring the early closure of the colostomy. Post-discharge management depends on growth status at birth and growth in the early postnatal period up to discharge. Preterm infants are susceptible to early iron deficiency and late vitamin D deficiency, and supplementation and monitoring are indicated. Our patient had iron deficiency (<12 mcg/L) at 6 weeks corrected age, and the neonatologist supplemented the diet with iron. The symptoms of early iron deficiency in preterm infants are the following: poor growth, gastrointestinal disturbances, thyroid dysfunction, susceptibility to infections, instability in temperature, and abnormal neurological reflexes with 37 weeks [40,41].

### 3.4. Long-Term Follow-Up and Neurodevelopmental Outcomes

Long-term neurodevelopmental assessment at 6 months chronological age shows appropriate milestones for corrected age. The infant demonstrates normal tone and reflexes, with no obvious neurological sequelae from extreme prematurity or surgical interventions. Continued monitoring through specialized follow-up programs remains essential [42].

### 3.5. Study Limitations

This case report has several limitations that should be acknowledged. Genetic testing was performed to rule out megacystis-microcolon syndrome, but specific ACTG2 and MYH11 mutations were not tested due to the limited availability of comprehensive genetic panels, which we recommend for future cases to better characterize the underlying pathophysiology. Long-term neurodevelopmental outcomes are limited to 6 months of chronological age, insufficient to detect subtle cognitive or motor deficits that may manifest later in childhood through extended follow-up programs. The nutritional assessment was based on clinical parameters and growth curves without detailed body composition analysis or specific micronutrient evaluations, limiting the understanding of precise nutritional challenges in this complex case. Maternal inflammatory markers and placental histopathological examination results were not systematically evaluated despite SARS-CoV-2 infection and prolonged membrane rupture, which could have provided valuable insights into the inflammatory cascade leading to extreme prematurity. This represents a single case from one institution, limiting the generalizability of the findings, as the successful outcome may reflect specific institutional expertise that may not be universally available, highlighting the need for multicenter studies with larger cohorts to establish evidence-based guidelines for managing similar complex cases.

To rescue these highly premature neonates, the maternity medical team’s efforts alone are insufficient; a multidisciplinary team, including a pediatric surgeon, is essential. Consequently, advancements in all facets of neonatal care have diminished infant morbidity and mortality, especially among the most vulnerable and critically ill infants.

## 4. Conclusions

This case demonstrates that extremely premature infants with rare gastrointestinal malformations can achieve favorable outcomes through early recognition, prompt multidisciplinary intervention, and coordinated long-term care. Key success factors include maintaining high clinical suspicion for unusual complications, willingness to pursue surgical intervention at very low birth weights when indicated, and comprehensive follow-up addressing both growth and neurodevelopmental outcomes.

## Figures and Tables

**Table 1:** Key clinical milestones during the initial hospitalization.

Timeline	Complication/Intervention	Management
Day 1	Respiratory distress, intubation	HFO ventilation, surfactant
Day 3	Patent ductus arteriosus (PDA)	Fluid restriction, IV paracetamol
Day 5	Ureaplasma suspected	Added clarithromycin
Day 10	Leukocytosis	Changed to imipenem/cilastatin, colistin
Day 12	Intestinal obstruction suspected	Surgical consultation

## Data Availability

The data can be shared upon request due to privacy concern.
